# Spatial localisation of Discoidin Domain Receptor 2 (DDR2) signalling is dependent on its collagen binding and kinase activity

**DOI:** 10.1016/j.bbrc.2018.04.191

**Published:** 2018-06-18

**Authors:** Maciej T. Luczynski, Peter T. Harrison, Nadia Lima, Lukas Krasny, Angela Paul, Paul H. Huang

**Affiliations:** a Division of Molecular Pathology, The Institute of Cancer Research, London, United Kingdom; b Proteomics Core Facility, The Institute of Cancer Research, London, United Kingdom

**Keywords:** Discoidin domain receptor, Signal transduction, Receptor tyrosine kinase, Collagen, DDR2

## Abstract

Discoidin Domain Receptor 2 (DDR2) is a collagen-binding receptor tyrosine kinase that initiates delayed and sustained tyrosine phosphorylation signalling. To understand the molecular basis of this unique phosphorylation profile, here we utilise fluorescence microscopy to map the spatiotemporal localisation of DDR2 and tyrosine phosphorylated proteins upon stimulation with collagen. We show that cellular phosphorylated proteins are localised to the interface where DDR2 is in contact with collagen and not in the early endosomes or lysosomes. We find that DDR2 localisation is independent of integrin activation and the key DDR2 signalling effector SHC1. Structure-function analysis reveals that DDR2 mutants defective for collagen binding or kinase activity are unable to localise to the cell surface, demonstrating for the first time that both collagen binding and kinase functions are required for spatial localisation of DDR2. This study provides new insights into the underlying structural features that control DDR2 activation in space and time.

## 1 Introduction

The Discoidin Domain Receptors (DDRs), comprising DDR1 and DDR2, are collagen-binding receptor tyrosine kinases (RTKs) that function as microenvironmental sensors at the interface of the extracellular matrix and the intracellular signal transduction machinery [1]. In response to ligand engagement, the DDRs undergo receptor tyrosine phosphorylation and recruit SH2- and PTB-containing adaptor proteins which trigger downstream signalling [2]. The DDRs are typified by a unique delayed and sustained tyrosine phosphorylation signalling profile. In contrast to growth factor activated RTKs where receptor phosphorylation is normally initiated within seconds to minutes of ligand binding and subsequently undergoes signal degradation by negative feedback regulation, the DDRs are activated by collagen with a delayed timescale of minutes to hours and tyrosine phosphorylation signalling remains sustained for several hours to days [3,4]. The mechanistic basis of this distinctive phosphorylation signalling dynamics is unknown, although it has been suggested that receptor localisation may be important [5]. Unlike other RTK family members such the ErbB, c-Met or PDGFR receptors, the spatiotemporal localisation of the DDRs remains poorly characterised.

A previous study employed Förster resonance energy transfer (FRET) imaging and fluorescence microscopy to determine the cellular location of DDR1 in HEK293 cells [6]. The authors of this study found that DDR1 is rapidly internalised within 5 min of collagen presentation. A very recent study utilized a combination of fluorescence microscopy and biochemistry to demonstrate that DDR1 undergoes ligand-induced clustering at the cell surface through lateral dimer association in COS-7 cells [7]. It is unknown if DDR2 displays a similar spatiotemporal profile as DDR1.

Here we undertake the first characterisation of the spatiotemporal localisation of DDR2 and its activation of cellular tyrosine phosphorylated proteins. Utilising immunofluorescence microscopy, we demonstrate for the first time that cellular tyrosine phosphorylated proteins are localised to the interface where DDR2 is in contact with collagen; and perform a series of biochemical experiments that delineate the key structural and signalling features that are necessary for this observed receptor localisation. This study provides new details into the fundamental requirements that regulates DDR2 activation in space and time.

## 2 Materials and methods

### 2.1 Cell culture and derivation of stable cell lines

Flp-In T-REx-293 cells (Thermofisher Scientific, Waltham, MA, USA) were cultured in DMEM media supplemented with 10% Tet-free FBS/2 mM glutamine/100 units/ml penicillin/100 mg/ml streptomycin in 95% air/5% CO_2_ atmosphere at 37 °C. For expression of DDR2, Flp-In T-REx-293 cells were transfected with pcDNA5/FRT/TO expression vector (Thermofisher Scientific) engineered with human cDNA for DDR2 [4] and pOG44 plasmid (Thermofisher Scientific) using the calcium phosphate method and selected in 100 μg/ml Hygromycin B (Invivogen, San Diego, CA, USA). Selected cells were pooled and analysed for DDR2 expression by immunoblotting analysis. Expression of DDR2 was induced by the addition of 1 μg/ml doxycycline 72 h prior to experiments (Sigma-Aldrich, St. Louis, MI, USA). Generation of DDR2 mutant, SHC1 shRNA knockdown cell lines and SILAC pulse experiments are described in Supplemental Methods.

### 2.2 Immunoblotting

DDR2 expression was induced by 1 μg/ml doxycycline for 72 h prior to serum starvation. Cells were stimulated at the indicated times with 20 μg/ml of acid-soluble rat tail collagen I #C766-25 mg (Sigma-Aldrich) or 2 mM acetic acid (Sigma-Aldrich) and lysed in RIPA lysis buffer (50 mM Tris HCl, pH7.6; 150 mM NaCl; 1% IGEPAL CA-630, 0.1% SDS; 0.5% sodium deoxycholate) supplemented with protease and phosphatase inhibitors (Thermofisher Scientific) at 4 °C. Lysates were loaded onto SDS-PAGE gels prior to blotting onto PVDF membranes (Thermofisher Scientific). Blots were probed with primary antibodies followed by corresponding horseradish peroxidase-conjugated (HRP) secondary antibodies. Details of antibodies used are provided in the Supplemental Methods. Immunoreactive bands were visualized by chemiluminescence (Thermofisher Scientific) and the blots were exposed to CL-XPosure Film (Thermofisher Scientific).

### 2.3 Confocal microscopy and immunofluorescence

Cells were seeded on μ-Slide 8-well chambered coverslips #80826 (Ibidi, Planegg, Germany) that were coated with 20 μg/ml poly-l-Lysine (Sigma-Aldrich). DDR2 expression was induced by 1 μg/ml doxycycline for 72 h prior to serum starvation. Cells were serum-starved overnight before stimulation with 20 μg/ml of acid-soluble rat tail collagen I (Sigma-Aldrich) or acid vehicle at the indicated time points, fixed with 4% formaldehyde for 15 min, permeabilised with 0.2% Triton-X 100/PBS for 10 min and then blocked with IF buffer (3% BSA, 0.05% Tween 20 in PBS) for 1 h. Specimens were incubated for 3 h with primary antibodies (as described in the Supplemental Methods) at 4 °C, rinsed five times with IF buffer and then incubated with secondary antibodies anti-rabbit Alexa488 and anti-goat Alexa555 (Thermofisher Scientific). DNA was visualised by DAPI (Thermofisher Scientific) staining. All confocal microscopy was performed on a Ziess LSM 710 microscope with the 60x NA oil objective at optimal aperture settings. For each field of view a z-stack of 20 images was captured. To visualise collagen I, acid-soluble rat tail collagen I (Sigma-Aldrich) was spiked with FITC conjugate-collagen Type I (Sigma-Aldrich) in 1:1 ratio. For P5D2-blocking experiments, cells were pre-treated with 10 μg/ml of anti-human Integrin β1 P5D2 #MAB17781 (R&D Systems), for 30 min before stimulation with collagen for 24 h. Image analysis methodology is described in the Supplemental Methods.

### 2.4 ELISA (enzyme-linked immunosorbent assay)

DDR2 expression was induced by 1 μg/ml doxycycline for 72 h prior to serum starvation. Cells were serum-starved overnight before stimulation with 20 μg/ml acid-soluble rat tail collagen I (Sigma-Aldrich) or acid control at the time points indicated and lysed in a lysis buffer (1% NP-40 Alternative, 20 mM Tris (pH 8.0), 137 mM NaCl, 10% glycerol, 2 mM EDTA) supplemented with protease and phosphatase inhibitors (Thermofisher Scientific). ELISA for phospho-DDR2 was performed using the Human Phospho-DDR2 DuoSet IC ELISA kit (R&D Systems). The reaction was visualized by the addition of 100 μl of chromogenic substrate reagent #DY999 (R&D Systems) for 20 min and the reaction was stopped with 50 μl of 1 M H_2_SO_4_ (Sigma-Aldrich). Absorbance at 450 nm was measured using a Spectra Max M5 plate reader (Molecular Devices, Sunnyvale, CA, USA).

## 3 Results

### 3.1 Mapping the localisation of DDR2-mediated tyrosine phosphorylation

**Fig. 1:**
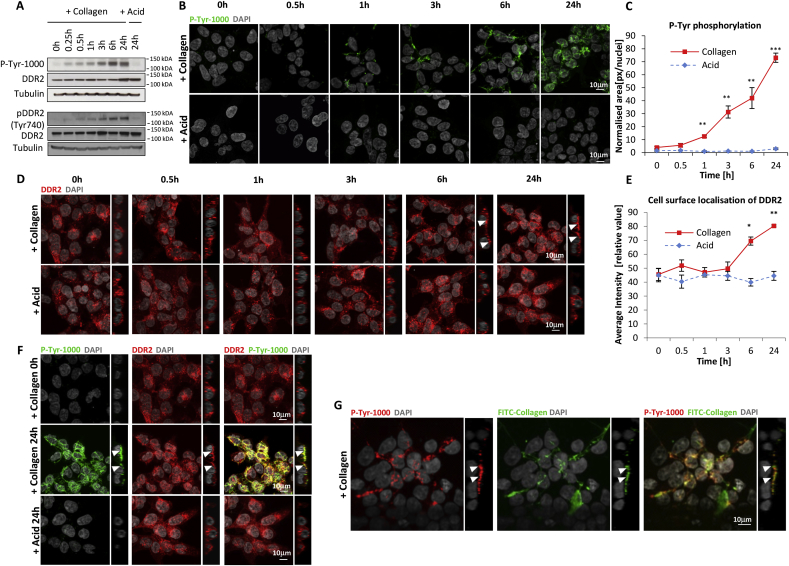
**DDR2 is tyrosine phosphorylated and localises to the cell surface in a collagen-dependent manner.** (A) Immunoblot showing the time course of DDR2 activation after stimulation with 20 μg/ml collagen I or acid control in DDR2-Flp-In T-REx HEK293 cells. Phosphotyrosine (pY) levels were measured by the P-Tyr-1000 antibody (upper panel) and receptor phosphorylation by DDR2 pY740 antibody (lower panel). (B) Panel of representative immunofluorescence images showing phosphotyrosine-containing proteins in DDR2-Flp-In T-REx HEK293 cells after stimulation with 20 μg/ml collagen I, DAPI (grey) and P-Tyr-1000 (green). (C) Plot depicting quantified phosphotyrosine immunofluorescence signal. Area occupied by P-Tyr-1000 antibody (green) was quantified using Image J and normalized by nuclei counts (n = 4). Values are means ± S.E.M. with ***p < 0.001 and **p < 0.01, indicating a significant difference between collagen I versus acid treatments as determined by paired Student's *t*-test. (D) Panel of representative images showing subcellular localisation of DDR2 receptor (red) in DDR2-Flp-In T-REx HEK293 cells after collagen I stimulation at different time points. Images represent z- and orthogonal projections. White arrows in the images with orthogonal projections indicate localisation of DDR2 at the cell surface, nuclei are shown in grey. (E) Plot depicting quantified enrichment of the DDR2 receptor at the cell surface after collagen I or acid stimulation in different time points (n = 4). Values are means ± S.E.M. with **p < 0.01 and *p < 0.05, indicating a significant difference between collagen I versus acid treatments at different time-points as determined by paired Student's *t*-test. (F) Representative images showing co-localisation (yellow) of DDR2 receptor (red) at the cell surface or phosphotyrosine-containing proteins (green) after collagen I stimulation versus acid control at 24 h. Images represent z- and orthogonal projections. White arrows in the images with orthogonal projections indicate localisation of phosphotyrosine-containing proteins and DDR2 at the cell surface. Full time course is presented in Fig. S4 (G) Representative images showing co-localisation (yellow) of FITC-conjugated collagen I (green) and phosphotyrosine-containing proteins (red) at the cell-surface interface after 24 h. Images represent z- and orthogonal projections. White arrows in the images with orthogonal projections indicate localisation of phosphotyrosine-containing proteins and collagen at the cell-surface interface. (For interpretation of the references to colour in this figure legend, the reader is referred to the Web version of this article.)

DDR2 was engineered into the Flp-In T-REx HEK293 cell line and evaluation of DDR2 induction in these engineered cells showed maximal receptor expression after 24 h of doxycycline exposure (Fig. S1). For all subsequent experiments in this study, DDR2-Flp-In T-REx HEK293 cells were treated for at least 48 h with doxycycline prior to stimulation with collagen. Stimulation of these cells with acid-soluble collagen I resulted in the activation of DDR2 tyrosine phosphorylation with the characteristic delayed and sustained kinetics as previously described (Fig. 1A) [4].

We sought to first define the cellular location of tyrosine phosphorylated proteins in the DDR2 expressing cells upon ligand stimulation. Cells were treated with collagen I over 6 time-points (0–24 h), and tyrosine phosphorylated proteins were visualised using immunofluorescence staining with a pan-specific anti-phosphotyrosine antibody P-Tyr-1000 (Fig. 1B). Protein tyrosine phosphorylation was observed beginning at 1 h post-stimulation with total phosphorylation levels increasing to a maximum at 24 h (Fig. 1B–C). As expected acid control-treated cells showed no tyrosine phosphorylation. Interestingly, the tyrosine phosphorylation staining initiated by collagen I displayed a fibrillar appearance (Fig. 1B). Performing a similar time-course experiment using an antibody for the phosphorylated Y740 residue in the activation loop of DDR2 recapitulated a similar pattern of staining (Fig. S2) [4], confirming that the tyrosine phosphorylation events measured by P-Tyr-1000 are a good surrogate for receptor-mediated signalling.

We next examined the localisation of DDR2 in the presence of collagen I or acid control. Immunofluorescence staining for total DDR2 shows that a proportion of DDR2 receptor is present at the cell surface interface at 6 and 24 h post-collagen stimulation which was not observed in the acid-treated controls (Fig. 1D–E). Stable isotope labelling with amino acids in cell culture (SILAC)-pulse analysis of DDR2 expression levels measured by mass spectrometry demonstrates that this increase in cell surface receptor levels is not due to differences in net receptor turnover between the acid and collagen treatments (Fig. S3) [8].

When collagen I was administered for 24 h, DDR2 and P-Tyr-1000 immunofluorescence signals showed strong co-localisation (Fig. 1F and Fig. S4). The fibrillar appearance of the P-Tyr-1000 staining was reminiscent of previously reported images of DDR2-mediated fibrillogenesis of exogenously added FITC-labelled soluble collagen I [9,10]. To determine if tyrosine phosphorylation was associated with collagen localisation at the cell-surface interface, we performed co-localisation experiments with P-Tyr-1000 and FITC-labelled collagen I. Phosphotyrosine staining showed a strong co-localisation with exogenously added collagen after 24 h (Fig. 1G), indicating that tyrosine phosphorylation triggered by DDR2 activation is localised to the interface where DDR2 is in contact with collagen.

Further examination of the subcellular location of DDR2 with markers of the early endosome (EEA1) and lysosome (LAMP1) compartments finds that a proportion of the receptor co-localises with the early endosomes (Fig. S5A) but not the lysosomes (Fig. S5B) both in the absence and presence of collagen. However, P-Tyr-1000 staining did not co-localise with either EEA1 and LAMP1 staining (Fig. S5) when the cells were stimulated with collagen, indicating that tyrosine phosphorylated proteins are not present within the early endosomes and lysosomes.

### 3.2 Collagen-mediated tyrosine phosphorylation and DDR2 localisation is independent of integrin activation

**Fig. 2:**
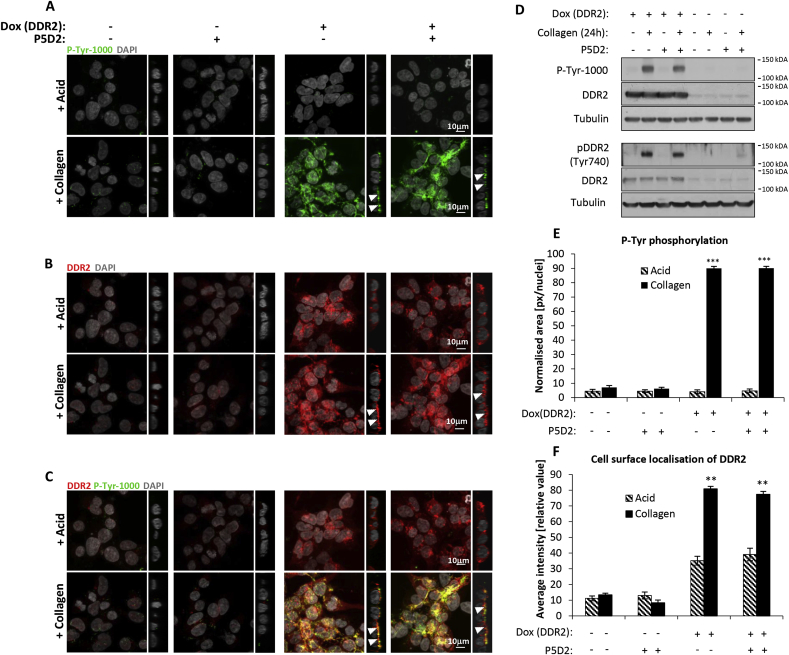
**Tyrosine phosphorylation and DDR2 cell surface localisation is independent of integrin activation.** Representative set of immunofluorescence images showing (A) phosphotyrosine-containing proteins, (B) DDR2 localisation and (C) merged images in DDR2-Flp-In T-REx HEK293 cells with and without induction of DDR2 expression by doxycycline (Dox). Cells were stimulated with 20 μg/ml collagen I for 24 h in the presence or absence of pre-treatment with P5D2 blocking antibody prior to collagen addition. DAPI (grey), P-Tyr-1000 (green), DDR2 (red) and co-localisation (yellow). Images represent z- and orthogonal projections. White arrows in the images with orthogonal projections indicate localisation of immunofluorescence signal at the cell surface. (D) Immunoblot showing activation of DDR2 in DDR2-Flp-In T-REx HEK293 cells ± doxycycline (Dox) induction of DDR2 after collagen I stimulation (20 μg/ml, 24 h) and cells ± pre-treatment with P5D2 blocking antibody prior to collagen I stimulation. Phosphotyrosine (pY) levels were measured by the P-Tyr-1000 antibody (upper panel) and receptor phosphorylation by DDR2 pY740 antibody (lower panel). (E) Bar plots showing quantified phosphotyrosine-containing proteins based on immunofluorescence images. Area occupied by P-Tyr-1000 antibody (green) was quantified and normalized by nuclei counts (n = 4). Values are means ± S.E.M. with ***p < 0.001, indicating a significant difference between collagen I versus acid control treatment as determined by paired Student's *t*-test. (F) Bar plots showing quantified enrichment of the DDR2 receptor at the cell surface after collagen I or acid control treatment for 24 h based on immunofluorescence images (n = 4). Values are means ± S.E.M. with **p < 0.01, indicating a significant difference between collagen I versus acid control treatment as determined by paired Student's *t*-test. (For interpretation of the references to colour in this figure legend, the reader is referred to the Web version of this article.)

The other major class of collagen-binding receptors are the integrins which are also known to activate tyrosine phosphorylation signalling [11,12]. We have previously shown that the HEK293 cells express endogenous levels of α1β1 and α2β1 collagen-binding integrins [4,13]. To exclude the possibility that the observed tyrosine phosphorylation events in our imaging experiments are due to activation of the integrins by collagen I, we conducted the immunofluorescence experiments in the absence of doxycycline induction of DDR2. In the absence of DDR2 expression, no tyrosine phosphorylation was observed when cells were stimulated with collagen I for 24 h using both imaging and immunoblotting methods, demonstrating that integrin activation is not the driver of the observed P-Tyr-1000 tyrosine phosphorylation signals (Fig. 2A, D, and E). In addition, we also stimulated DDR2 expressing cells with collagen I in the presence of the integrin β1 functional blocking antibody P5D2 which prevents collagen binding to the α1β1 and α2β1 integrins [13,14]. Blocking integrin activation did not alter tyrosine phosphorylation levels (Fig. 2A, D and E), cell surface localisation of DDR2 (Fig. 2B and F) or co-localisation of DDR2 with P-Tyr-1000 staining in the presence of collagen I at 24 h (Fig. 2C). Collectively, these data confirm that tyrosine phosphorylation and localisation of DDR2 is specific to DDR2 activation by collagen I and independent of integrin activity.

#### 3.2.1 SHC1 is not required for DDR2 phosphorylation and cell surface localisation

SHC1 is a key validated downstream effector of DDR2 signalling and is essential for multiple *in vivo* biological functions such as axon regeneration [[15], [16], [17]]. To assess the importance of SHC1 in regulating DDR2 localisation and phosphorylation, we generated shRNA SHC1 knockdowns in the DDR2-Flp-In T-REx HEK293 cells.

**Fig. 3:**
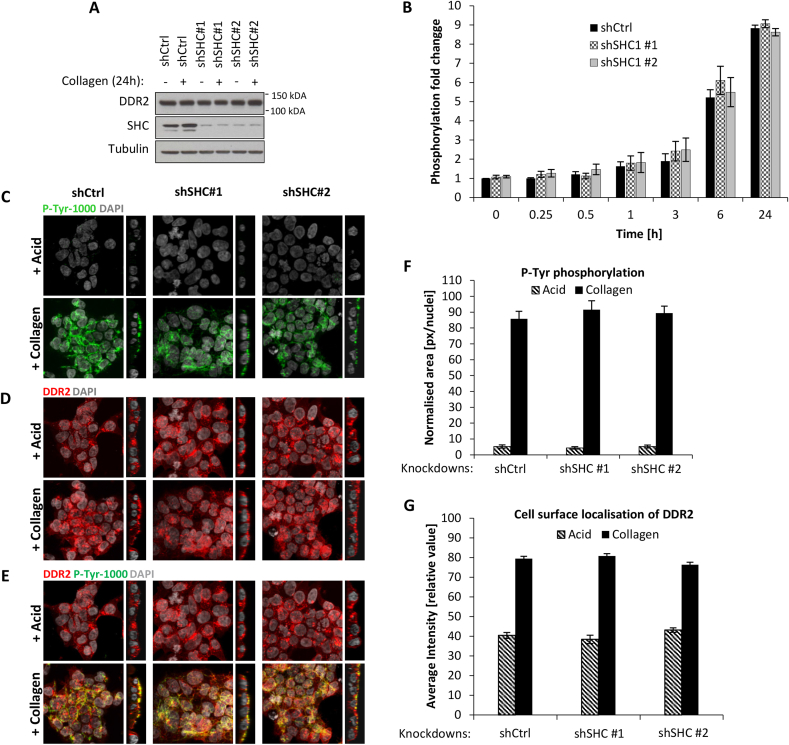
**SHC1 is not required for DDR2 phosphorylation and cell surface localisation.** (A) Immunoblot confirming SHC1 protein level depletion in shRNA knockdown experiments. Two distinct shRNA constructs were used (shSHC1#1 and shSHC1#2) in DDR2-Flp-In T-REx HEK293 cells. shCtrl represent the shRNA control cells. (B) ELISA measurements of DDR2 tyrosine phosphorylation in shCtrl and shSHC1 cells post-stimulation with collagen I at different time points (n = 4). Values are means ± S.E.M. Representative set of immunofluorescence images showing (C) phosphotyrosine-containing proteins, (D) DDR2 localisation and (E) merged images in DDR2-Flp-In T-REx HEK293 cells with SHC shRNA knockdown. Cells were stimulated with 20 μg/ml collagen I for 24 h. DAPI (grey), P-Tyr-1000 (green), DDR2 (red) and co-localisation (yellow). Images represent z- and orthogonal projections. (F) Bar plots showing quantification loss of phosphotyrosine-containing proteins based on immunofluorescence images. Area occupied by P-Tyr-1000 antibody (green) was quantified and normalized by nuclei count (n = 4). Values are means ± S.E.M. (G) Bar plots showing quantified enrichment of DDR2 at the cell surface after collagen I or acid control treatment for 24 h based on immunofluorescence images (n = 4). Values are means ± S.E.M. (For interpretation of the references to colour in this figure legend, the reader is referred to the Web version of this article.)

Silencing of SHC1 in the DDR2 expressing cells was confirmed by immunoblotting (Fig. 3A). Assessment of DDR2 tyrosine phosphorylation by ELISA measurements finds that SHC1 depletion did not have an effect on DDR2 phosphorylation levels across multiple timepoints (0–24 h) post-stimulation with collagen I compared to control cells (Fig. 3B). Immunofluorescence analysis showed that SHC1 loss did not alter protein tyrosine phosphorylation levels (Fig. 3C and F), DDR2 localisation at the cell surface (Fig. 3D and G) or co-localisation of DDR2 with P-Tyr-1000 staining in the presence of collagen I (Fig. 3E). These data demonstrate that SHC1 is dispensable for the regulation of DDR2 receptor localisation and phosphorylation dynamics.

#### 3.2.2 DDR2 cellular localisation is dependent on its collagen binding and kinase activity

**Fig. 4:**
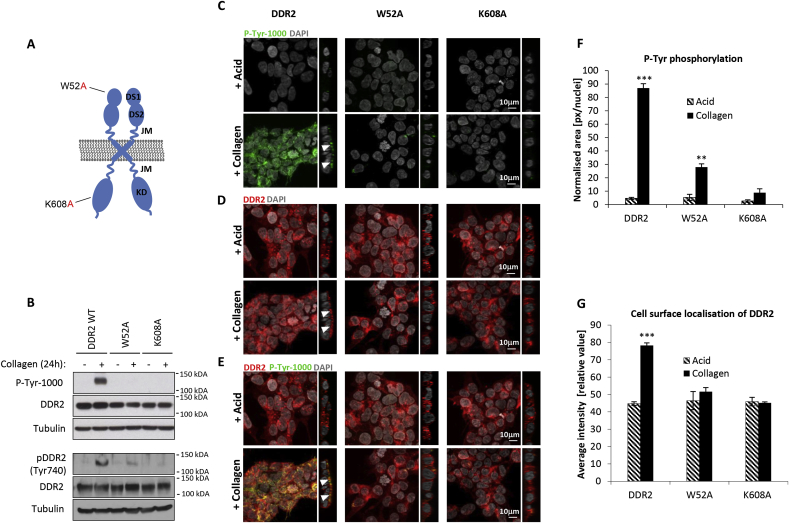
**DDR2 localisation to the cell surface requires both its collagen binding function and kinase activity.** (A) Domain organization and location of DDR2 point mutations used in this study. DS1, discoidin domain; DS2, discoidin-like domain; JM, juxtamembrane; KD, kinase domain. W52A is the collagen binding defective mutant and K608A is the kinase dead mutant. (B) Immunoblot showing loss of DDR2 activation in the W52A and K608A mutants. Phosphotyrosine (pY) levels were measured by the P-Tyr-1000 antibody (upper panel) and receptor phosphorylation by DDR2 pY740 antibody (lower panel). Representative set of immunofluorescence images showing (C) phosphotyrosine-containing proteins, (D) DDR2 localisation and (E) merged images in Flp-In T-REx HEK293 cells expressing DDR2, W52A or K608E mutant stimulated with 20 μg/ml collagen I for 24 h. DAPI (grey), P-Tyr-1000 (green), DDR2 (red) and co-localisation (yellow). Images represent z- and orthogonal projections. White arrows in the images with orthogonal projections indicate localisation of immunofluorescence signal at the cell surface. (F) Bar plots showing quantified loss of phosphotyrosine-contain proteins for DDR2, W52A and K608A mutants based on immunofluorescence images. Area occupied by P-Tyr-1000 antibody (green) was quantified and normalized by nuclei counts (n = 4). Values are means ± S.E.M. with ***p < 0.001 and **p < 0.01, indicating a significant difference between collagen I versus acid control as determined by paired Student's *t*-test. (G) Bar plots showing quantified enrichment of DDR2, W52A and K608A at the cell surface after collagen I or acid control treatment for 24 h based on immunofluorescence images (n = 4). Values are means ± S.E.M. with ***p < 0.001, indicating a significant difference between collagen I versus acid control treatment as determined by paired Student's *t*-test. (For interpretation of the references to colour in this figure legend, the reader is referred to the Web version of this article.)

We posited that collagen binding may be a requirement for the localisation of DDR2 to the cell surface in the presence of its ligand. To test this hypothesis, we undertook a structure-function analysis using a DDR2 functional mutant defective for collagen binding. The amino acid residues in the amphiphilic collagen-binding pocket of the discoidin-homology (DS) domain in DDR2 are highly conserved and a W52A mutation in the binding pocket reduces DDR2 collagen binding capacity (Fig. 4A) [[18], [19], [20]]. We engineered DDR2 W52A into the Flp-In T-Rex HEK293 cell line which showed a slight decrease in total receptor expression levels compared to the wildtype DDR2-expressing cells (Fig. 4B). Immunofluorescence analysis in the presence of collagen or acid control for 24 h showed that as expected, the W52A mutant had a diminished ability for activating tyrosine phosphorylation compared to the wildtype receptor (Fig. 4B, C, E and F). Analysis of the localisation of DDR2 by immunofluorescence found that unlike the wildtype receptor, the W52A mutant did not localise to the cell surface in the presence of collagen, maintaining a similar receptor distribution as acid-treated control cells (Fig. 4D and G).

The other major role of DDR2 is a receptor tyrosine kinase. It has been shown that the collagen binding activity of DDR2 is maintained in kinase-deficient forms of the receptor, indicating that the collagen binding properties of the receptor are independent of its kinase function [9]. To determine if kinase activity is required for the receptor localisation in the presence of collagen, we generated a kinase-dead version of DDR2 (K608A) which harbours an inactivating mutation in the catalytic lysine of the kinase domain (Fig 4A) [4]. Similar to the W52A mutant, cells expressing DDR2 K608A show a slight reduction in total receptor expression levels compared to wildtype (Fig 4B). As anticipated, the kinase-dead mutant showed no tyrosine phosphorylation upon stimulation with collagen I (Figure 4B, C, E and F). Unexpectedly, this K608A mutant also did not localise at the cell surface 24 h post-collagen stimulation, displaying a similar phenotype as the acid-treated controls (Fig 4D and G). Taken together, our findings demonstrate that both the collagen binding and kinase activities of DDR2 are necessary for receptor localisation at the cell surface in the presence of collagen.

## 4 Discussion

In this study, we provide the first spatiotemporal characterisation of DDR2 localisation and tyrosine phosphorylation signalling. Our imaging data demonstrates that upon ligand presentation, a proportion of cellular DDR2 is localised to the cell surface. The localisation of DDR2 and cellular phosphorylated proteins is independent of the collagen-binding integrins and the presence of SHC1. Our experiments also find that tyrosine phosphorylated proteins are present at the interface where DDR2 is in contact with collagen and not in the early endosomes or lysosomes. Importantly we show that an intact collagen binding pocket is necessary for the spatiotemporal localisation of DDR2 at the cell surface, providing the first evidence that collagen binding is a key driver for receptor localisation. Since the collagen binding activity of DDR2 is independent of its kinase function [9], our data also led to the unexpected and novel finding that kinase activity is necessary for receptor localisation at the cell surface.

In this study, we were able derive new insights into the biology of DDR2 including the demonstration that cellular tyrosine phosphorylated proteins co-localise with DDR2 and exogenously added collagen I (Fig. 1G). This finding suggests that similar to the focal adhesion complexes associated with integrin activation by collagen [21], DDR2 signalling is a spatially localised event occurring at discrete locations at the cell-surface interface where it is in close proximity with the collagen ligand.

Taken together, our imaging data on DDR2 and the body of work on DDR1 trafficking supports the view that the DDRs are localised to the cell surface where these receptors bind to collagen and induce tyrosine phosphorylation [6,7,22,23]. By utilising immunofluorescence microscopy, this study reveals the spatial heterogeneity associated with DDR2 phosphorylation-mediated signalling and the functional components required for receptor localisation, ultimately uncovering new insights into DDR2 signalling in both space and time.

## Conflicts of interest

Authors declare no conflicts of interest.

## Appendix A Supplementary data

Supplemental Fig. Legend Supplemental Fig. Legend

Supplemental Methods docx Supplemental Methods docx
