# Corrigendum to “Spatial localisation of Discoidin Domain Receptor 2 (DDR2) signalling is dependent on its collagen binding and kinase activity” [Biochem. Biophys. Res. Commun. 501 (1) (18 June 2018) 124–130]

**DOI:** 10.1016/j.bbrc.2018.08.051

**Published:** 2018-09-26

**Authors:** Maciej T. Luczynski, Peter T. Harrison, Nadia Lima, Lukas Krasny, Angela Paul, Paul H. Huang

**Affiliations:** a Division of Molecular Pathology, The Institute of Cancer Research, London, United Kingdom; b Proteomics Core Facility, The Institute of Cancer Research, London, United Kingdom

The authors regret that they have made a minor error in their article.

The mass range of DDR2 in the western blot in supplemental figure S1 was mislabelled as 75–100 kDa. The correct mass range should be 100 kDa-150kDa.

**Figure:**
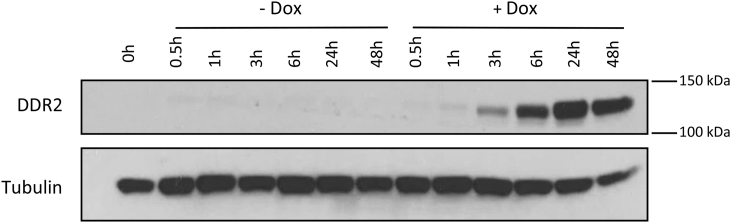

This error does not change the conclusions of the paper.

The authors would like to apologise for any inconvenience caused.

