# Supplementary figures and images for: Spatial localisation of Discoidin Domain Receptor 2 (DDR2) signalling is dependent on its collagen binding and kinase activity
Source: Biochem Biophys Res Commun. 2018 Jun 18;501(1):124–30. doi: 10.1016/j.bbrc.2018.04.191 (PMC5964065; doi:10.1016/j.bbrc.2018.04.191)

## Slide 1
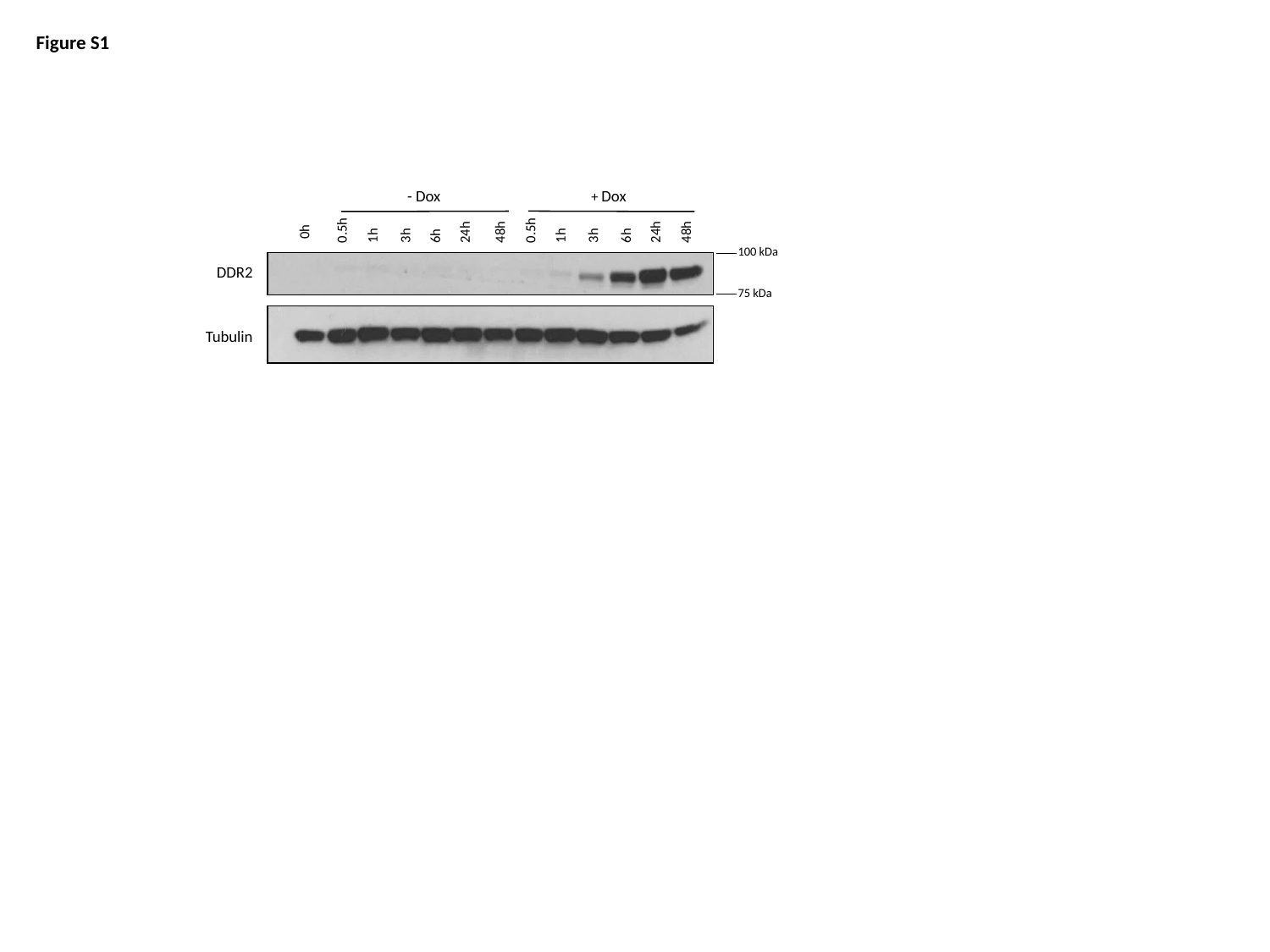

Figure S1
- Dox
+ Dox
0h
0.5h
1h
3h
6h
24h
48h
0.5h
1h
3h
6h
24h
48h
100 kDa
DDR2
75 kDa
Tubulin

Supplement: Fig. S1 [file mmc1.pptx]

## Slide 1
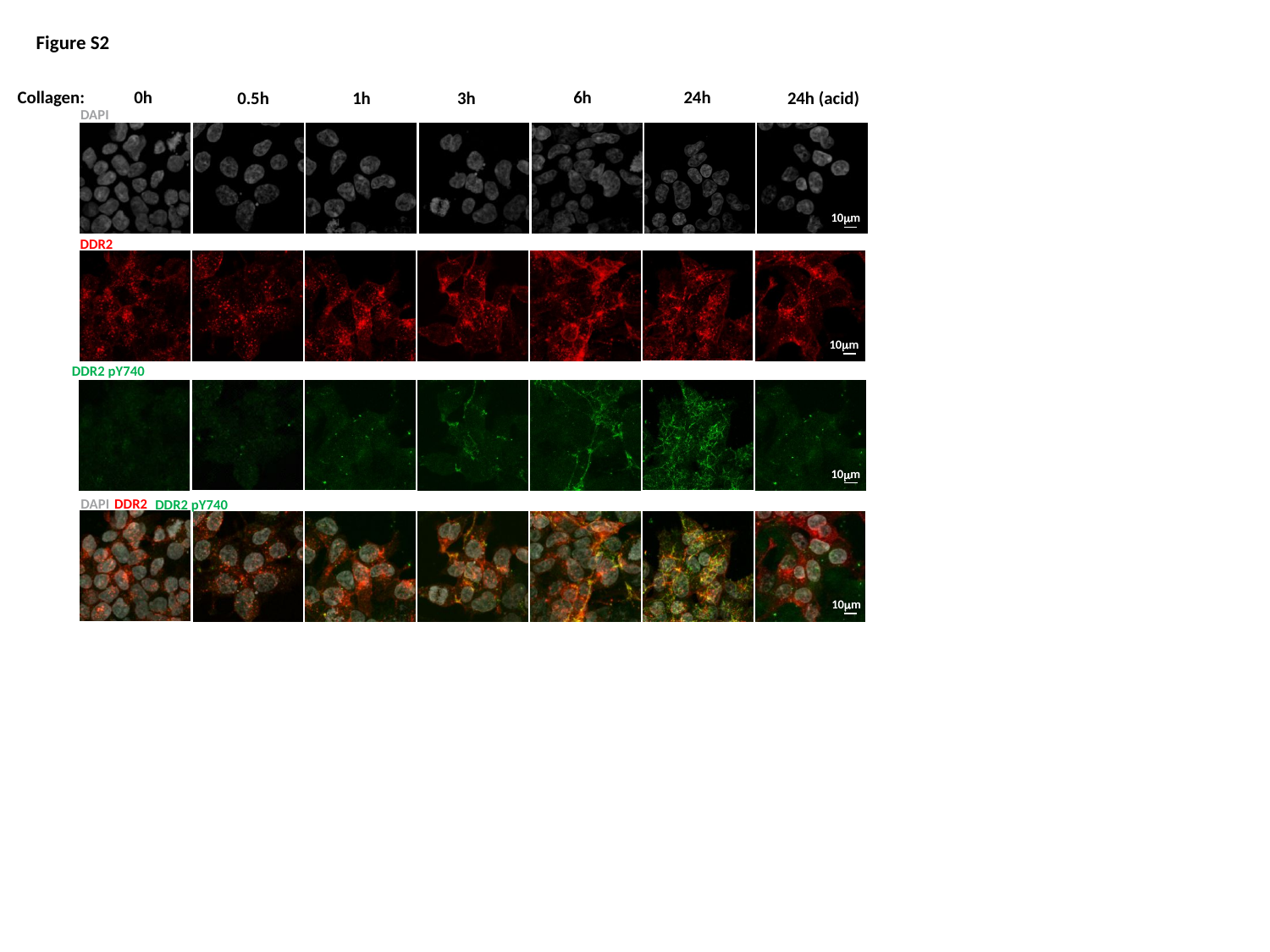

Figure S2
Collagen:
24h
6h
0h
24h (acid)
3h
1h
0.5h
DAPI
10mm
DDR2
10mm
DDR2 pY740
10mm
DDR2
DAPI
 DDR2 pY740
10mm

Supplement: Fig. S2 [file mmc2.pptx]

## Slide 1
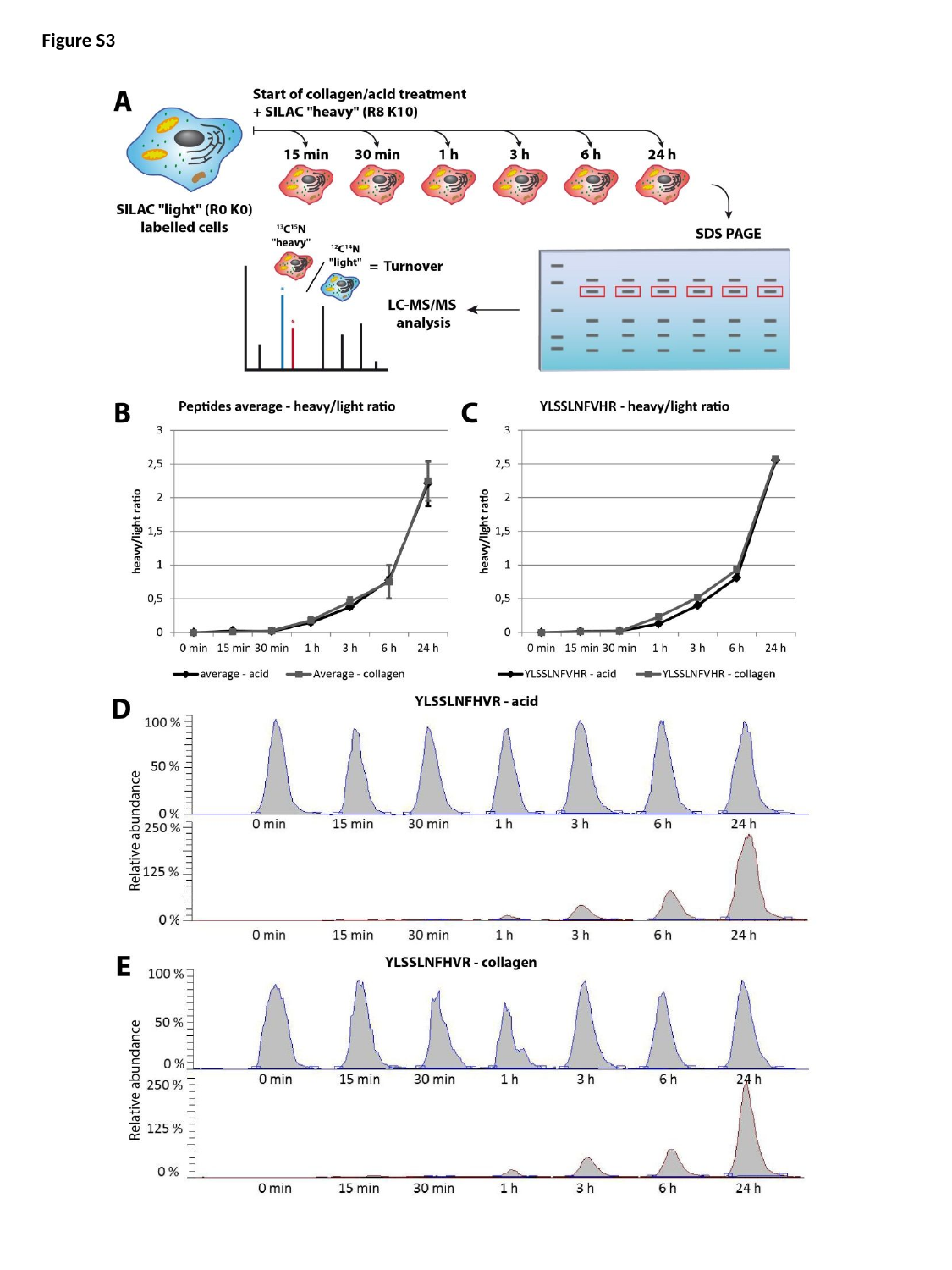

Figure S3

Supplement: Fig. S3 [file mmc3.pptx]

## Slide 1
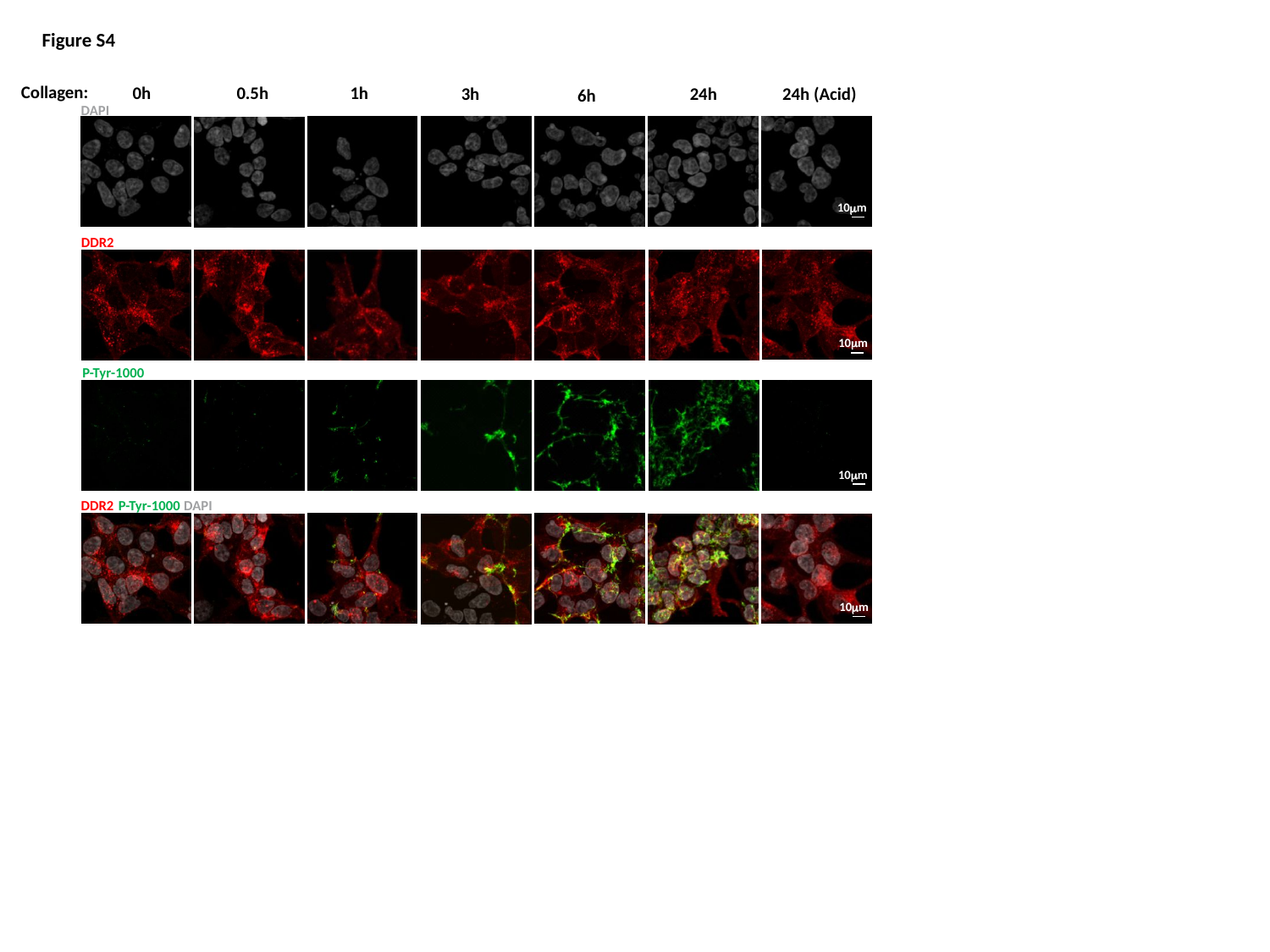

Figure S4
Collagen:
1h
0.5h
0h
3h
24h (Acid)
24h
6h
DAPI
10mm
DDR2
10mm
P-Tyr-1000
10mm
DDR2
DAPI
P-Tyr-1000
10mm

Supplement: Fig. S4 [file mmc4.pptx]
