# Supplementary material for: Spatial localisation of Discoidin Domain Receptor 2 (DDR2) signalling is dependent on its collagen binding and kinase activity
Source: Biochem Biophys Res Commun. 2018 Jun 18;501(1):124–30. doi: 10.1016/j.bbrc.2018.04.191 (PMC5964065; doi:10.1016/j.bbrc.2018.04.191)
Supplement: Fig. S5 [file mmc5.pptx]

## Slide 1
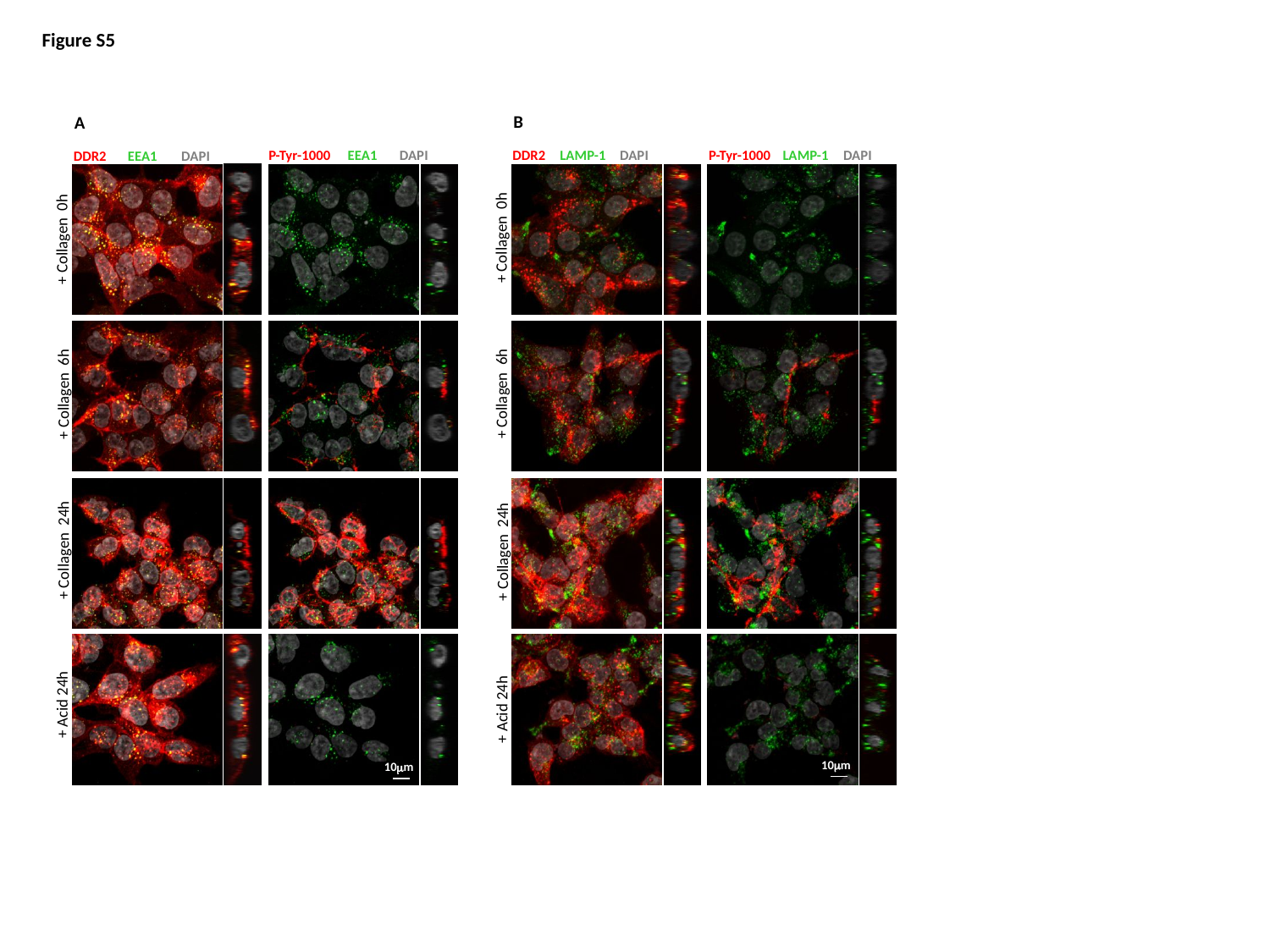

Figure S5
B
A
P-Tyr-1000
LAMP-1
DAPI
P-Tyr-1000
EEA1
DAPI
DDR2
LAMP-1
DAPI
DDR2
EEA1
DAPI
+ Collagen 0h
+ Collagen 0h
+ Collagen 6h
+ Collagen 6h
+ Collagen 24h
+ Collagen 24h
+ Acid 24h
+ Acid 24h
10mm
10mm
