# Supplementary material for: Spatial localisation of Discoidin Domain Receptor 2 (DDR2) signalling is dependent on its collagen binding and kinase activity
Source: Biochem Biophys Res Commun. 2018 Jun 18;501(1):124–30. doi: 10.1016/j.bbrc.2018.04.191 (PMC5964065; doi:10.1016/j.bbrc.2018.04.191)
Supplement: Supplemental Fig. Legend [file mmc6.docx]

**Supplemental figure legends**

**Figure S1. Immunoblot of inducible DDR2 expression in DDR2-Flp-In T-REx HEK293 cells.** Cells were treated with 1µg/ml doxycycline (Dox) or vehicle for the indicated time points prior to lysis. Maximal receptor expression is achieved after 24 hours of doxycycline exposure.

**Figure S2. Immunofluorescence imaging of total DDR2 and DDR2 pY740 in DDR2-Flp-In T-REx HEK293** **cells.** Panel of representative immunofluorescence images showing phosphotyrosine-containing proteins in DDR2-Flp-In T-REx HEK293 cells after stimulation with 20 μg/ml collagen I at the indicated time points. DAPI (grey), DDR2 (Red), pY740 DDR2 (green) and co-localisation (yellow).

**Figure S3. SILAC pulse analysis of DDR2 net turnover.** (A) SILAC pulse method, DDR2-Flp-In T-REx HEK293 cultured in light SILAC media (blue) is subjected to collagen I or acid treatment with media changed to heavy SILAC medium (heavy). Cells are then harvested and lysed at different times. Lysates are separated on SDS-PAGE with bands corresponding to the molecular weight of DDR2 excised and subjected to LC-MS/MS analysis. The H/L ratio at each time point measures the net DDR2 protein turnover. (B) Averaged quantification of net DDR2 protein turnover across three distinct DDR2 peptides (YSSLNFVHR, DVAVEEFPR and DLEPPIVAR) across 7 timepoints (0 – 24 hours) after collagen I or acid stimulation shows that there is no difference in the net DDR2 turnover between the two conditions. (C) Quantification of exemplar DDR2 peptide YSSLNFVHR across 7 timepoints (0 – 24 hours) after collagen I or acid stimulation. Extracted precursor ion chromatograms for SILAC light (blue) and heavy (red) variants of the YSSLNFVHR peptide across 7 timepoints in (D) acid or (E) collagen I treatment demonstrates similar incorporation of heavy amino acids in both acid and collagen I conditions.

**Figure S4. Immunofluorescence imaging of total DDR2 and P-Tyr-1000 in DDR2-Flp-In T-REx HEK293 cells.** Panel of representative immunofluorescence images showing phosphotyrosine-containing proteins in DDR2-Flp-In T-REx HEK293 cells after stimulation with 20 μg/ml collagen I at the indicated time points. DAPI (grey), DDR2 (Red), P-Tyr-1000 (green) and co-localisation (yellow) at the indicated time points.

**Figure S5. Localisation of DDR2 and tyrosine phosphorylated proteins in the early endosome and lysosome compartments.** (A) Representative images showing localisation of left panel - DDR2 receptor (red), right panel – P-Tyr-1000 (red), early endosome marker – EEA1 (green) or co-localisation (yellow) after collagen I stimulation at the indicated time points versus acid control at 24 hours. Images represent z- and orthogonal projections. (B) Representative images showing localisation of left panel - DDR2 receptor (red), right panel – P-Tyr-1000 (red) and lysosomal-associated membrane protein 1 - LAMP1 (green) after collagen I stimulation at different time points versus acid control at 24 hours. Images represent z- and orthogonal projections.
