# Supplementary material for: Spatial localisation of Discoidin Domain Receptor 2 (DDR2) signalling is dependent on its collagen binding and kinase activity
Source: Biochem Biophys Res Commun. 2018 Jun 18;501(1):124–30. doi: 10.1016/j.bbrc.2018.04.191 (PMC5964065; doi:10.1016/j.bbrc.2018.04.191)
Supplement: Supplemental Methods docx [file mmc7.docx]

**Supplemental Methods**

*Generation of DDR2 mutants and SHC1 shRNA knockdown cells.*

DDR2 mutants were generated using the QuikChange® XL site-directed mutagenesis kit (Stratagene, San Diego, CA, USA) according to the manufacturer's protocol. Primers used for the mutagenesis are detailed below: W52A: 5'-cacagcttccagtcaggcgtcagagtccacagctg-3' and 5’-cagctgtggactctgacgcctgactggaagctgtg-3’; K608A: 5'- tgtcctggtggctgtggcaatgctccgagcagatg-3’ and 5’-catctgctcggagcattgccacagccaccaggaca-3'. Successful site-directed mutagenesis was confirmed by sequencing (Eurofins, Luxembourg).

SHC1 shRNA stable knockdowns were generated using the MISSION shRNA constructs (Sigma-Aldrich). Knockdowns were prepared with two separate shRNA constructs (SHC1: TRCN0000040208, TRCN0000040212). Control cell line was generated using shRNA sequence targeting eGFP (#SHC005). Lentiviral particles were generated in 293T cells that were co-transfected with pKLO (Sigma-Aldrich), pMD2.G and psPAX2 (Addgene, Cambridge, MA, USA) lentiviral DNA vectors using Lipofectamine 2000 (Thermofisher Scientific). Lentiviral infection of Flp-In T-Rex-293 cells was carried out aiming to transduce about 80% of the total amount of cells in each experiment, using an MOI of 10. To select for infected cells, puromycin (Thermofisher Scientific) was added to the media to a final concentration of 1µg/mL and the selection process was carried out for 10 days. Selected cells were pooled and efficiency of knockdowns was analysed by immunoblotting.

*Antibodies*

Primary antibodies include anti-human DDR2 #AF2538, anti-human integrin β1 #MAB17781 (R&D Systems, Minneapolis, MN, USA), anti-SHC #610878 (BD Biosciences, San Jose, CA, USA), P-Tyr-1000 #8954 (Cell Signaling Technologies, Danvers, MA, USA), pY740 #DDR2_p47 (rabbit serum, custom order [[30](#_ENREF_30)]), anti-EEA1 #610456 (BD Biosciences), or anti-LAMP-1 #MAB4800 (R&D systems) and anti-α-Tubulin #T5168, (Sigma-Aldrich). Secondary antibodies include anti-goat HRP #43C-CB0527-FIT (Stratech Scientific), anti-rabbit HRP #7074 (Cell Signaling Technologies) and anti-mouse HRP #G32-62G-1000 (Signalchem, Richmond, BC, Canada).

*Image analysis*

Image analysis was performed with Fiji software ([www.fiji.sc](http://www.fiji.sc)). To quantify phosphotyrosine signalling from the images, a z-stack was converted into a z-projection. To measure the area occupied by the phosphotyrosine signal, projections were thresholded to eliminate background signal and each field of view was normalised by number of nuclei. Average values from three analysed fields of view were considered as one biological replicate. Four biological replicates were performed per experiment.

To quantify enrichment of DDR2 at the cell surface: 1. the total area occupied by the DDR2 receptor was thresholded to reduce background signal; 2. average intensity of anti-goat Alexa555 antibody was measured in every single image within a z-stack of 20 images; and 3. average intensities measured from 5 images corresponding to localisation at the cell surface were bound together and plotted. Average values from three analysed fields of view were considered as one biological replicate. Each experiment was run in 4 biological replicates.

*SILAC pulse experiment*

For preparation of lysates, DDR2-Flp-In T-REx HEK293 cells were seeded into T75 flasks with 1µg/ml doxycycline to induce DDR2 expression and allowed to attach for 72 h. Cells were then serum starved for 16 h. After washing with heavy SILAC media, cells were stimulated with either 20 µg/ml collagen I or 100 mM acetic acid in fresh heavy SILAC media for 15 min, 30 min, 1 h, 3 h, 6 h and 24 h. Treated cells were lysed at the indicated timepoints in 500µl cold RIPA lysis buffer.

Equal amounts of lysates from each time point were separated by SDS-PAGE and bands with the molecular weight corresponding to DDR2 were excised from the gel and digested as described in [[31](#_ENREF_31)]. Briefly, gel bands were cut into small cubes, then destained and dehydrated in 50% acetonitrile, 50mM triethylammonium bicarbonate (TEAB), reduced by 10 mM tris(2-carboxyethyl)phosphine (TCEP) (40 min at 56°C) and alkylated by 55mM iodacetamide (30 min at 25°C in dark). After washing and dehydration in 50% acetonitrile, 50mM TEAB, MS sequencing grade trypsin (12.5ng/µl) in 50mM TEAB was added to digest protein and incubated overnight at 37 °C. Digestion was stopped by acidification to 0.5 % (v/v) TFA. Then, peptides were extracted by 50% acetonitrile, 0.1% TFA and desalted by ZipTip C18 (Merck Millipore Ltd. Cork, Ireland) according to manufacturer’s protocol, prior to LC-MS/MS analysis.

For LC-MS/MS analysis, peptides were dissolved in Buffer A (2% acetonitrile, 0.1% formic acid) and separated on UltiMate 3000 RSLCnano (Thermofisher Scientific) system using a 50 cm C18 Easy-Spray packed emitter column (2 μm particle size; PepMap RSLC, Thermo Fisher Scientific) with  a multi-step gradient of buffers A:B (4-10% B, t = 20 min 10-25% B, t = 100 min 25-50% B, t = 125 min, 10-95% B, t = 130 min, 95% B, t = 135 min. buffer A: 2% acetonitrile/0.1% formic acid; buffer B: 80% acetonitrile/0.1% formic acid) with a flow rate of 250 nl/min. MS/MS spectra were acquired on Thermo Q Exactive HF (Thermofisher Scientific) Orbitrap mass spectrometer in positive mode. Acquired raw spectra were searched against the human sequences present in the Uniprot_sprot database using Proteome Discoverer 2.0 software (Thermofisher Scientific) with a parent ion mass tolerance of 15 ppm and fragment ion mass tolerance of 0.02 Da. Scaffold 4.4.6 (Proteome Software Inc. Portland, OR, USA) was used to validate MS/MS based peptide and protein identifications. Extraction of precursor ion chromatograms for heavy and light variants of identified DDR2 peptides was performed by Xcalibur (Thermo Fisher Scientific) software with mass tolerance of 15 ppm. Generated chromatograms were smoothed by sampled Gaussian kernel with 7 sampling points and area under the curve of the peaks was integrated by Xcalibur using default method. Heavy-to-light ratio of peak areas for individual peptides and time points was calculated and plotted using Microsoft Excel 2010.
